# Minimal Models of Multidimensional Computations

**DOI:** 10.1371/journal.pcbi.1001111

**Published:** 2011-03-24

**Authors:** Jeffrey D. Fitzgerald, Lawrence C. Sincich, Tatyana O. Sharpee

**Affiliations:** 1 Computational Neurobiology Laboratory, The Salk Institute for Biological Studies, La Jolla, California, United States of America; 2 Center for Theoretical Biological Physics and Department of Physics, University of California, San Diego, La Jolla, California, United States of America; 3 Beckman Vision Center, University of California, San Francisco, San Francisco, California, United States of America; University College London, United Kingdom

## Abstract

The multidimensional computations performed by many biological systems are often characterized with limited information about the correlations between inputs and outputs. Given this limitation, our approach is to construct the maximum noise entropy response function of the system, leading to a closed-form and minimally biased model consistent with a given set of constraints on the input/output moments; the result is equivalent to conditional random field models from machine learning. For systems with binary outputs, such as neurons encoding sensory stimuli, the maximum noise entropy models are logistic functions whose arguments depend on the constraints. A constraint on the average output turns the binary maximum noise entropy models into minimum mutual information models, allowing for the calculation of the information content of the constraints and an information theoretic characterization of the system's computations. We use this approach to analyze the nonlinear input/output functions in macaque retina and thalamus; although these systems have been previously shown to be responsive to two input dimensions, the functional form of the response function in this reduced space had not been unambiguously identified. A second order model based on the logistic function is found to be both necessary and sufficient to accurately describe the neural responses to naturalistic stimuli, accounting for an average of 93% of the mutual information with a small number of parameters. Thus, despite the fact that the stimulus is highly non-Gaussian, the vast majority of the information in the neural responses is related to first and second order correlations. Our results suggest a principled and unbiased way to model multidimensional computations and determine the statistics of the inputs that are being encoded in the outputs.

## Author Summary

Biological systems across many scales, from molecules to ecosystems, can all be considered information processors, detecting important events in their environment and transforming them into actions. Detecting events of interest in the presence of noise and other overlapping events often necessitates the use of nonlinear transformations of inputs. The nonlinear nature of the relationships between inputs and outputs makes it difficult to characterize them experimentally given the limitations imposed by data collection. Here we discuss how minimal models of the nonlinear input/output relationships of information processing systems can be constructed by maximizing a quantity called the noise entropy. The proposed approach can be used to “focus” the available data by determining which input/output correlations are important and creating the least-biased model consistent with those correlations. We hope that this method will aid the exploration of the computations carried out by complex biological systems and expand our understanding of basic phenomena in the biological world.

## Introduction

There is an emerging view that the primary function of many biological systems, from the molecular level to ecosystems, is to process information [1]–[4]. The nature of the computations these systems perform can be quite complex [5], often due to large numbers of components interacting over wide spatial and temporal scales, and to the amount of data necessary to fully characterize those interactions. Constructing a model of the system using limited knowledge of the correlations between inputs and outputs can impose implicit assumptions and biases leading to a mischaracterization of the computations. To minimize this type of bias, we maximize the noise entropy of the system subject to constraints on the input/output moments, resulting in the response function that agrees with our limited knowledge and is maximally uncommitted toward everything else. An equivalent approach in machine learning is known as conditional random fields [6]. We apply this idea to study neural coding, showing that logistic functions not only maximize the noise entropy for binary outputs, but are also special closed-form cases of the minimum mutual information (MinMI) solutions [7] when the average firing rate of a neuron is fixed. Recently, MinMI was used to assess the information content in constraints on the interactions between neurons in a network [8]. We use this idea to study single neuron coding to discover what statistics of the inputs are encoded in the outputs. In macaque retina and lateral geniculate nucleus, we find that the single neuron responses to naturalistic stimuli are well described with only first and second order moments constrained. Thus, the vast majority of the information encoded in the spiking of these cells is related only to the first and second order statistics of the inputs.

To begin, consider a system which at each moment in time receives a 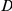-dimensional input 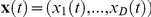 from a known distribution 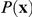, such as a neuron receiving a sensory stimulus or post-synaptic potentials. The system then performs some computation to determine the output 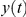 according to its response function 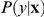. The complete input/output correlation structure, i.e. all moments involving 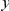 and 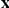, can be calculated from this function through the joint distribution 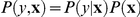, e.g. 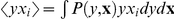. Alternatively, the full list of such moments contains the same information about the computation as the response function itself, although such a list is infinite and experimentally impossible to obtain. However, a partial list is usually obtainable, and as a first step we can force the input/output correlations from the model to match those which are known from the data. The problem is then choosing from the infinite number of models that agree with those constraints. Following the argument of Jaynes [9], [10], we seek the model which avails the most uncertainty about how the system will respond.

Information about the identity of the input can be obtained by observing the output, or vice versa, quantified by the mutual information 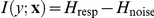 [11], [12]. The first term is the response entropy, 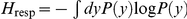, which captures the overall uncertainty in the output. The second term is the so-called noise entropy [13],
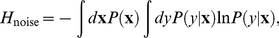
(1)representing the uncertainty in 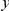 that remains if 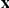 is known. If the inputs completely determine the outputs, there is no noise and the mutual information reaches its highest possible value, 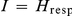. In many realistic situations however, repeated presentations of the same inputs produce variable outputs producing a nonzero noise entropy [14] and lowering the information transmitted.

By maximizing the noise entropy, the model is forced to be consistent with the known stimulus/response relationships but is as uncertain as possible with respect to everything else. We show that this maximum noise entropy (MNE) response function for binary output systems with fixed average outputs is also a minimally informative one. This approach is a special closed-form case of the mutual information minimization technique [8], which has been used to address the information content of constraints on the interactions between neurons. Here we use the minimization of the mutual information to characterize the computations of single neurons and discover what about the stimulus is being encoded in their spiking behavior.

## Results

### Maximum noise entropy models

The starting point for constructing any maximum noise entropy model is the specification of a set of constraints 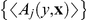, where 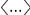 indicates an average over the joint distribution 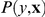. These constraints reflect what is known about the system from experimental measurements, or a hypothesis about what is relevant for the information processing of the system. For neural coding, the constraints could be quantities such as the spike-triggered average [15]–[18] or covariance [19]–[22], equivalent to 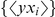 and 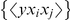, respectively. With each additional constraint, our knowledge of the true input/output relationship increases and the correlation structure of the model becomes more similar to that of the actual system.

Given the constraints, the general MNE response function is given by (see Methods)
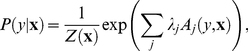
(2)where the 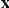-dependent partition function 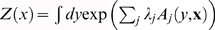 ensures that the MNE response function is consistent with normalization, i.e. 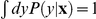. The MNE response function in Eq. (2) has the form of a Boltzmann distribution [23] with a Lagrange multiplier 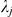 for each constraint. The values of these parameters are found by matching the experimentally observed averages with the analytical averages obtained by from derivatives of 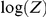 [23].

### Binary responses and minimum mutual information

**Figure 1:**
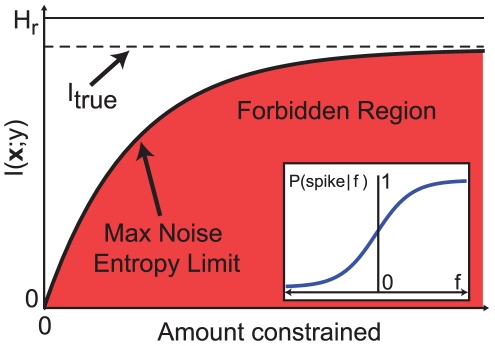
The maximum noise entropy (MNE) limit. This cartoon illustrates the consequences of a minimally informative, MNE response function. As knowledge of the correlation structure increases (which amounts to constraining more moments of the conditional output distribution), the least possible amount of information consistent with that knowledge increases along the solid line. Below the MNE limit is a forbidden region where a response function cannot be consistent with the given set of constraints. All models are bounded from above by the response entropy, corresponding to a noiseless system. Any response function above the MNE limit thus involves unknown and unconstrained moments which carry information. The information associated with the MNE response function increases toward the true value as the knowledge of the distribution tends to infinity. For a binary system, the response function is a logistic function (inset) in the transformed input space defined by 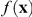, cf. Eq. (3).

Many systems in biological settings produce binary outputs. For instance, the neural state 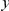 can be thought of as binary, with 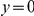 for the silent state and 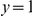 for the “spiking” state, during which an action potential is fired [13]. The inputs themselves could be a sensory stimulus or all of the synaptic activity impinging upon a neuron, both of which are typically high-dimensional [24]. Another example is gene regulation [25], where the inputs could be the concentrations of transcription factors and the binary output represents an on/off transcription state of the gene. For these systems, the constraints of interest are proportional to 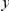. This is because any moments independent of 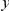 will cancel due to the partition function and any moments with higher powers are redundant, e.g. 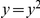 if 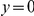 or 1. In this case, the set of constraints may be written more specifically as 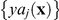 and the MNE response function becomes the well-known logistic function
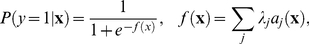
(3)with 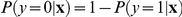. Thus for all binary MNE models, the effect of the constraints is to perform a nonlinear transformation of the input variables, 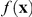, to a space where the spike probability is given by the logistic function (inset, Fig. 1).

For neural coding, one of the most fundamental and easily measured quantities is the total number of spikes produced by a neuron over the course of an experiment, equivalent to the mean firing rate. By constraining this quantity, or more specifically its normalized version 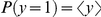, the MNE model is turned into a minimum information model. This holds because the response entropy 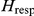 is completely determined by the distribution 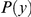, which is in turn constrained by 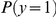 if the response is binary. With the response entropy constrained to match the experimentally observed system, maximizing the noise entropy is equivalent to minimizing information. Therefore, as was proposed in [7], any model that satisfies a given set of constraints will convey the information that is due only to those constraints. With each additional constraint our knowledge of the correlation structure increases along with the minimum possible information given that knowledge, which approaches the true value as illustrated schematically in Fig. 1.

The simplest choice is a first order model (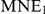) where the spikes are correlated with each input dimension separately. This model requires knowledge of the set of moments 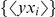, the spike-triggered average stimulus. For 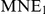, the transformation on the inputs is linear, 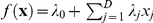, where the constant 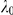 is the Lagrange multiplier for the spike probability constraint. With knowledge of only first order correlations, we see that the model neuron is effectively one-dimensional, choosing a single dimension in the 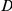-dimensional input space 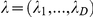 and disregarding all information about any other directions.

With higher order constraints, the transformation is nonlinear and the model neuron is truly multidimensional. For instance, the next level of complexity is a second order model (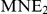), in which spikes may also interact with pairs of inputs. This model is obtained by constraining 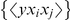, equivalent to knowing the spike-triggered covariance of the stimulus, resulting in the input transformation 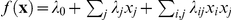. Any other MNE model can be constructed in the same fashion by choosing a different set of constraints, reflecting different amounts of knowledge.

**Figure 2:**
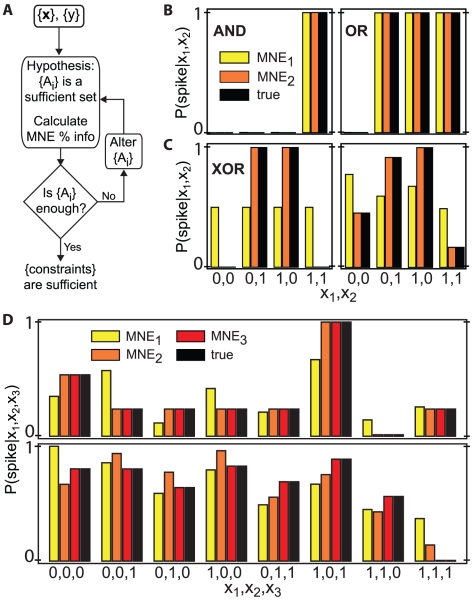
Using the MNE method for response functions to binary inputs. **A**) Flowchart representing how to determine the relevant constraints. The hypothesis that a minimal set of constraints is sufficient is tested by constructing the corresponding MNE model and calculating the information captured by the model. If the percent information is insufficient, the set of constraints is augmented. **B**) Response functions and MNE models for two binary inputs; the true system is shown in black, and first and second order MNE models (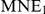 and 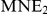) in yellow and orange, respectively. The AND and OR gates use only first order interactions; both MNE models explain 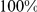 of the information. **C**) The XOR gate (left) uses only second order interactions; 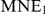 explains 0% while 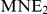 explains 100% of the information. An example of a mixed response function (right), for which both first and second order interactions are used (10% and 100% respectively). **D**) Two examples of response functions with three binary inputs, with 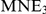 shown in red. Only second order interactions are necessary for the top gate, with 48%, 100% and 100% of the information captured by the first, second and third order MNE models. For the bottom gate, the models capture 39%, 71% and 100% of the information, indicating that third order constraints are necessary. In all cases, 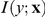 was calculated assuming a uniform input distribution.

The mutual information of the MNE model 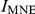 is the information content of the constraints. The ratio of 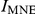 to the empirical estimate 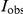 of the true mutual information of the system is the percent of the information captured by the constraints. This quantity is always less than or equal to one, with equality being reached if and only if all of the relevant moments have been constrained. This suggests a procedure to identify the relevant constraints, described in Fig. 2A. First, a hypothesis is made about which constraints are important. Then the corresponding MNE model is constructed and the information calculated. If the information captured is too small, the constraints are modified until a sufficiently large percentage is reached. Any constraints beyond that are relatively unimportant for describing the computation of the neuron.

As an illustrative example of the MNE method, consider a binary neuron which itself receives binary inputs (i.e. a logic gate). If the neuron in question receives 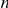 binary inputs, we are guaranteed to capture 100% of the information with 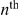-order statistics because all moments involving powers greater than one of either 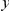 or any 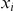 are redundant. However, different coding schemes may encode different statistics of the inputs. For instance, if the neuron receives only two inputs (Fig. 2B), the well-known AND and OR logic gate behaviors are completely described with only first order moments [26]. Correspondingly, the first order model 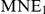 captures 100% of the information. Such a neuron can be said to encode only first order statistics of the inputs, and the spike-triggered average stimulus contains all of the information necessary to fully understand the computation. On the other hand, the XOR gate (Fig. 2C, left) requires second order interactions. This is reflected by 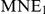 and 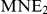 accounting for 0% and 100% of the information, respectively. More complicated coding schemes may involve both first and second order interactions, such as for the gate shown in the right panel of Fig. 2C. Here, 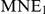 and 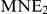 account for 10% and 100% of the information, respectively, and correctly quantify the degree to which each order of interaction is relevant to this neuron.

Similar situations show up for neurons that receive three binary inputs. The top panel of Fig. 2D shows an example of a neuron which only requires second order interactions. The parameters of 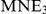 are exactly the same as 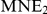, with the third order coefficient 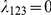. The bottom panel shows an example of a situation in which third order interactions are necessary. Correspondingly, 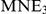 increases the information explained over 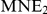 from 71% to 100%. These simulations demonstrate that despite the different coding schemes used by neurons, the information content of each order of interaction can be correctly identified using logistic MNE models.

### Neural coding of naturalistic inputs

In their natural environment, neurons commonly encode high-dimensional analog inputs, such as a visual or auditory stimulus as a function of time. It is important to note that the non-binary nature of the inputs means that the ability to capture 100% of the information between 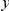 and the 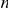 inputs with 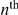-order statistics is not guaranteed anymore. Often, the dimensionality of the inputs may be reduced because the neurons are driven by a smaller subspace of relevant dimensions (e.g. [27]–[33]). However, even in those cases we are often forced to use qualitative terms such as ‘ring’ or ‘crescent’ to describe the experimentally observed response functions. With no principled way of fitting empirical response functions, the details of the interactions between neural responses and reduced inputs have been difficult to quantify.

The MNE method provides a quantitative framework for characterizing neural response functions, which we now apply to 9 retinal ganglion cells (RGCs) and 9 cells in the lateral geniculate nucleus (LGN) of macaque monkeys, recorded *in vivo* (see Methods). The visual input was a time dependent sequence of luminance values synthesized to mimic the non-Gaussian statistics of light intensity fluctuations in the natural visual environment [34]–[36].

**Figure 3:**
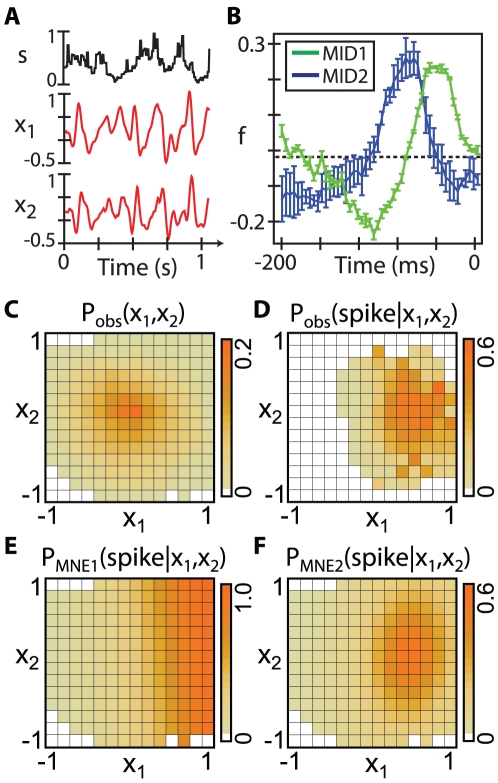
MNE models for a RGC. **A**) The normalized luminance 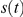 of the visual input, along with the two most informative reduced inputs, 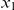 and 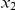, shown for a section of the stimulus presented to neuron mn122R4_3_RGC. **B**) The two maximally informative dimensions (MID) for this neuron (error bars are standard error in the mean). Each dimension is a filter which spans 200 ms before the neural output. The convolution of these filters with the stimulus produce 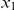 and 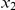, which are normalized to lie in the range -1 to 1. In this 2-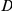 reduced input space, the input distribution, **C**), and observed response function, **D**), are shown, discretized into 14 bins along each dimension. White squares in the input distribution indicate unsampled inputs, while white squares in the response function indicate no spikes were recorded. The first order, **E**), and second order, **F**), MNE response functions for this cell explain 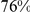 and 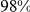 of the information, respectively.

A 1s segment of the normalized light intensity 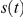 is shown in Fig. 3A. A previous study has shown that the responses of these neurons are correlated with the stimulus over an approximately 200 ms window preceding the response. When binned at 4 ms resolution, which ensures binary responses, the input is a vector in a 50 dimensional space. However, spikes are well predicted by using a 2 dimensional subspace [29] identified through the Maximally Informative Dimensions (MID) technique [37].

These two relevant dimensions, shown for a RGC in Fig. 3B, form a two dimensional receptive field which preserves the most information about the spikes in going from 50 to 2 dimensions. The two linear filters are convolved with the stimulus to produce reduced inputs 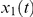 and 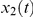, shown in Fig. 3A. The resulting input probability distribution in the reduced space is shown in Fig. 3C. The measured responses of the neuron then form a two-dimensional response function shown in Fig. 3D, where the color scale indicates the probability of a spike as a function of the two relevant input components.

To gain insight into the nature of this neuron's computational function and find the important interactions, we apply the MNE method starting with the first order MNE model shown in Fig. 3E. The first order model produces a response function which bears little resemblance to the empirical one and accounts for only 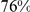 of the information. The next step is a second order MNE model (Fig. 3F), which produces a response function quite similar to the empirical one in both shape and amplitude, while accounting for 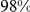 of the information. Thus, for this neuron, knowledge of second order moments is both necessary and sufficient to generate a highly accurate model of the neural responses.

**Figure 4:**
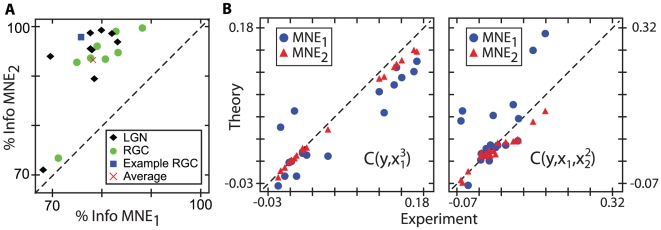
Second order MNE models are sufficient across the population. **A**) A direct comparison of the percent information captured by 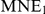 and 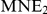. No cells are sufficiently modeled with a first order model, but most are with the second order model. The average information captured is 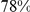 for 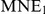 and 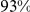 for 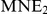. **B**) Comparison of experimentally measured values to theoretical predictions for higher-order unconstrained moments. Predictions for 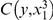, left, and 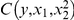, right, show a dramatic improvement when second order interactions are included in the model.

This result was typical across the population of cells, as illustrated in Fig. 4A by comparing the information captured by the first order versus second order models. The majority of the cells were well described by the second order model, accounting for over 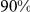 of the information. When averaged across the population, the first order model captured 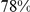 and the second order model captured 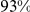 of 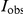. These results suggest that the inclusion of second order interactions are both necessary and sufficient to describe the responses of these neurons to naturalistic stimuli.

Since the MNE response function is a distribution of outputs given inputs, another way to check the effectiveness of any MNE model is to compare its moments with those obtained from experiments. The moments constrained to obtain the model will be identical to the experimental values by construction; it is the higher order moments, left unconstrained, that should be compared. In Fig. 4B we show two such comparisons for the correlation functions 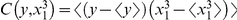 and 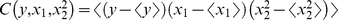, which involve moments unconstrained in the 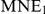 and 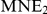 models. In both cases, the first order model predictions show more scatter than those of the second order model; the latter does a reasonable job of predicting the experimentally observed correlations. This result broadly demonstrates the sufficiency of second order interactions to model these neural responses, and shows that higher-order moments carry little to no additional information.

**Figure 5:**
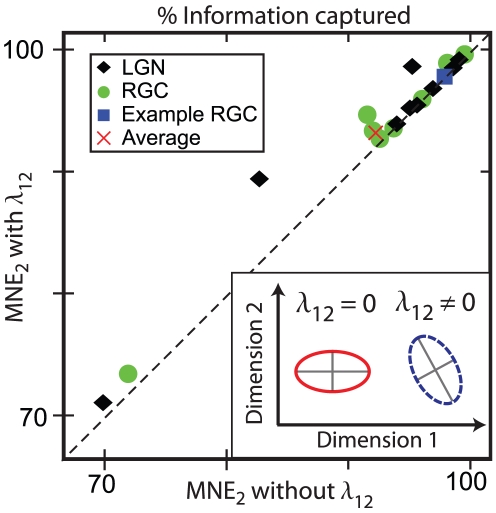
Importance of the mixed second order moments. A comparison of the percent of the information captured by a second order model (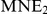) that constrains 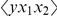 (i.e. 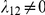) and a second order model with 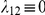. For most cells, the information increases only slightly for 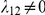, indicating that little information is gained by constraining this moment. (Inset) The parameter 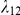 determines the angle between the axes of symmetry of the response function and the basis of the input space.

The two-dimensional second order MNE response functions have contours of constant probability which are conic sections. The parameter which governs the interaction between the two input dimensions, 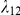, is related to the degree to which the axes of symmetry of the conic sections are aligned with the two-dimensional basis. For example, if the contours are ellipses, then 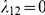 if the semi-major and semi-minor axes are parallel to the axes chosen to describe the input space, and 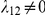 otherwise (see inset, Fig. 5). To assess the importance of this cross term, we compared the performance of second order MNE models with and without 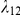. This additional term can only improve the performance of the model; however, as shown in Fig. 5, the improvements across the population are small. Thus, the dimensions found using the MID method are naturally parallel to the axes of symmetry of the response functions; however, this does not imply that the response function is separable due to the 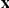 dependence of the normalization term 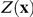.

## Discussion

For neural coding of naturalistic visual stimuli in early visual processing, we see that the bulk of what is being encoded is first order stimulus statistics. While the information gained by measuring the spike-triggered average is substantial, it is insufficient to accurately describe the neural responses. A second order model, which takes into account the spike-triggered input covariance, adds a sufficient amount of information. Thus the firing rates of these neurons have encoded the first and second order statistics of the inputs. Due to the fact that the natural inputs are non-binary and non-Gaussian, there exists a potential for very high-order interactions to be represented in the neural firing rate. It is known that higher order parameters of textures are perceptually salient [38]–[40], but it is unknown whether high order temporal statistics are also perceptually salient. Our results suggest that such temporal statistics are not encoded in the time-dependent firing rate, although they could be represented through populations of neurons or specific temporal sequences of spikes [41], [42].

Jaynes' principle of maximum entropy [9], [10] has a long and diverse history, with example applications in image restoration in astrophysics [43], extension of Wiener analysis to nonlinear stochastic transducers [44] and more recently in neuroscience [45]–[47]. In the latter studies, 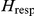 was maximized subject to constraints on the first and second order moments of the neural states 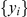 and 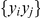 for a set of neurons in a network. The resulting pairwise Ising model was shown to accurately describe the distribution of network states 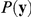 of real neurons under various conditions. Since then the application of the Ising model to neuroscience has received much attention [48], [49], and it is still a subject of debate if and how these results extrapolate to larger populations of neurons [50]. Temporal correlations have also been shown to be important in both cortical slices and networks of cultured neurons [47].

In contrast to maximum entropy models that deal with stationary or averaged distributions of states, the goal of maximizing the noise entropy is to find unbiased response functions. This approach is equivalent to conditional random field (CRF) models [6] in machine learning. The parameters of a CRF are fit by maximizing the likelihood using iterative or gradient ascent algorithms [51] and have been used, for example, in classification and segmentation tasks [52]. The parameters of MNE models may also be found using maximum likelihood, or as was done here, by solving a set of simultaneous constraint equations numerically. Another example of a maximum noise entropy distribution is the Fermi-Dirac distribution [23] from statistical physics, which is a logistic function governing the binary occupation of fermion energy levels. Thus, in the same way that the Boltzmann distribution was interpreted by Jaynes as the most random one consistent with measurements of the energy, the Fermi-Dirac distribution can be interpreted as the least biased binary response function consistent with an average energy. However, to our knowledge, this method has never been used in the context of neural coding to determine the input statistics which are being encoded by a neuron and create the corresponding unbiased models.

Previous work has applied the principle of minimum mutual information (MinMI) [7] to neural coding, thus identifying the relevant interactions between neurons [8]. We have shown that the closed-form MNE solutions for binary neurons constitute a special case of MinMI, since the response entropy is fixed if the average firing rate is constrained. In general, the MinMI principle results in a self-consistent solution that must be solved iteratively to obtain the response function. The reason why MNE models are closed-form is that the constraints are formulated in terms of moments of the output distribution instead of the output distribution itself. In addition to the case of binary responses, MNE models can become closed-form MinMI models for any input/output systems where the response entropy can be fixed in terms of the moments of the output variable. Examples include Poisson processes with fixed average response rate or Gaussian processes with fixed mean and variance of the response rate. The framework for analyzing the interactions between inputs and outputs that we present here can thus be extended to a broad and diverse set of computational systems.

Our approach can be compared to other optimization techniques commonly used to study information processing. For example, rate-distortion theory [11], [12], [53], [54] seeks minimum information transmission rate over a channel with a fixed level of signal distortion, e.g. lossy image or video compression. In that case, the best solution is the one which transmits minimal information because this determines the average length of the codewords. In our method, we also obtain minimally informative solutions, not because they are optimal for signal transmission, but because they are the most unbiased guess at a solution given limited knowledge of a complex system.

At the other end of the optimization spectrum is maximization of information [1], [13], [55]. The goal in that case is to study not how the neuron *does* compute, but how it *should* compute to get the most information, perhaps with limited resources. This strategy has been used to find neural response functions for single neurons [56], [57], as well as networks [58], [59]. When confronted with incomplete knowledge of the correlation structure, a maximum information approach would choose the values of the unconstrained moments such that they convey the most information possible, whereas the minimum information approach provides a lower bound to the true mutual information, and allows us to investigate how this lower bound increases as more moments are included. If the goal is to study the limits of neural coding, then maximizing the information may be the best procedure. If, however, the goal is to dissect the computational function of an observed neuron, we argue that the more agnostic approaches of maximizing the noise entropy or minimizing the mutual information are better-suited.

## Methods

### Ethics statement

Experimental data were collected as part of the previous study using procedures approved by the UCSF Institutional Animal Care and Use Committee, and in accordance with National Institutes of Health guidelines.

### Maximum noise entropy model

A maximum noise entropy model is a response function 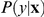 which agrees with a set of constraints and is maximally unbiased toward everything else. The constraints are experimentally observed moments involving the response 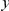 and stimulus 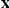, 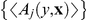, where 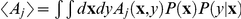, which must be reproduced by the model. The set of 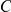 constraints, including the normalization of 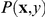, are then added to the noise entropy to form the functional
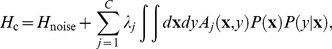
(4)with a Lagrange multiplier 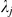 for each constraint. Setting 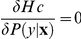 and enforcing normalization yields Eq. 1. For a binary system, 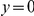 or 1, all the constraints take the form 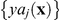, and the partition function is 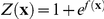, where 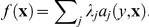


The values of the Lagrange multipliers are found such that the set of equations
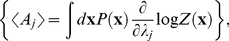
(5)is satisfied, with the analytical averages on the right-hand side obtained from derivatives of the free energy 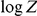 [23]. Simultaneously solving this set of equations has previously been shown to be equivalent to maximizing the log-likelihood [51].

### Physiology experiment

The neural data analyzed here were collected in a previous study [29] and the details are found therein. Briefly, the stimulus was a spot of light covering a cell's receptive field center, flickering with non-Gaussian statistics that mimic those of light intensity fluctuations found in natural environments [35], [36]. The values of light intensities were updated every 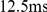 (update rate 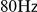). The spikes were recorded extracellularly in the LGN with high signal-to-noise, allowing for excitatory post-synaptic potentials generated by the RGC inputs to be recorded. From such data, the complete spike trains of both RGCs and LGN neurons could be reconstructed [60].

### Dimensionality reduction

The neural spike trains were binned at 4 ms resolution, ensuring that the response was binary. The stimulus was re-binned at 250 Hz to match the bin size of the spike analysis. The neurons were uncorrelated with light fluctuations beyond 200 ms before a spike, and the stimulus vector 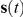 was taken to be the 200 ms window (50 time points) of the stimulus preceding 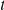. Just two projections of this 50-dimensional input are sufficient to capture a large fraction of the information between the light intensity fluctuations and the neural responses (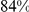 for the example neuron mn122R4_3_RGC, and 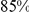 on average across the population). The two most relevant features of each neuron were found by searching the space of all linear combinations of two input dimensions for those which accounted for maximal information in the measured neural responses [37], subject to cross-validation to avoid overfitting. Each of the two features, 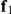 and 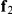, is a 50-dimensional vector which converts the input into a reduced input, calculated by taking the dot product, i.e. 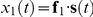. The algorithm for searching for maximally informative dimensions is available online at http://cnl-t.salk.edu.
